# Proceedings of Van Zandt Co. Med. Ass’n

**Published:** 1887-03

**Authors:** 


### PROCEEDINGS VANZANDT COUNTY MEDICAL ASSOCIATION.

The physicians of Van Zandt county, Texas, organized the Van Zandt County Medical Society, April 19, 1886. Dr. W. W. Reeves, of Wills Point, was elected President; Dr. J. I. D. Gray, Vice President; Dr. W. B. Watkins, Treasurer, and Dr. J. F. M. McCarty, Secretary.

We are indebted to Dr. McCarty, the well chosen Secretary, for full reports of all meetings of the Society to date; from them we extract the following interesting points, regretting that we have not space to publish the reports in full.

The Society was admitted to affilliation with the State Association at Dallas last April. Drs. Wakefield, Gray and Williams being the delegates chosen, with Drs. Reeves, Sisson and McCarty alternates. All the delegates were in attendance. The members take an active interest in the meetings, and some valuable papers have been read and discussed, amongst them, one by Dr. Reeves, the President, giving the history of a case of ovariotomy in his practice, which paper was ordered published.

Dr. Wakefield reported a case of prolapse of the cord at seventh month, with no history of rupture of the membranes. When the patient was placed in the recumbent position the cord would receed into the uterus, to fall again on resuming the erect posture. No satisfactory examination could be made

Dr. Johnson reported a case of alarming uterine hemorrhage at four months of gestation, due to a fibroid polypus two inches long, attached to inner surface of cervix. It was removed, and the patient went to full term. Dr. Johnson also reported a case where the cord having wound around the fœtus in such a way as to interfere with delivery, was tied and cut *in situ*, and the child was safely delivered.

The Van Zandt Medical Society made strenuous endeavors to have the law enforced against certain irregulars in the county, but the district attorney informed the committee that owing to defects in the law it is inoperative. Drs. Gray, Sisson and McCarty were appointed a committee to confer with the representatives from that district with a view to having the law so amended as to reach offenders. [We believe no effort has been made at this session of the Legislature to change or amend the law; it is a dead letter].

It is intensely gratifying to this JOURNAL to see so much interest manifested in the necessity of thorough organization of the profession. Many counties are now organized and in good working order, and hence the State Association is being rapidly built up. This is a kind of hobby with us, and to stimulate organization of local societies has been the chief mission of the JOURNAL—to which it has ever lent its best endeavors. If all the counties would do as Van Zandt and others have done, we would soon be able to influence legislation. It can only be done by united effort of the profession.

